# Risk Assessment for Tomato Fruitworm in Processing Tomato Crop-Egg Location and Sequential Sampling

**DOI:** 10.3390/insects12010013

**Published:** 2020-12-28

**Authors:** Elisabete Figueiredo, Catarina Gonçalves, Sónia Duarte, Maria C. Godinho, António Mexia, Laura Torres

**Affiliations:** 1 Linking Landscape, Environment, Agriculture and Food (LEAF), Instituto Superior de Agronomia, Universidade de Lisboa, 1349-017 Lisboa, Portugal; sduarte@isa.ulisboa.pt (S.D.); amexia@isa.ulisboa.pt (A.M.); 2 RAIZ—Instituto de Investigação da Floresta e Papel, Quinta de São Francisco, Apartado 15, 3801-501 Eixo-Aveiro, Portugal; catarina.goncalves@thenavigatorcompany.com; 3 Escola Superior Agrária de Santarém, Instituto Politécnico de Santarém, Quinta do Galinheiro, S. Pedro, 2001-904 Santarém, Portugal; maria.godinho@esa.ipsantarem.pt; 4 CITAB-Centre for the Research and Technology of Agro-Environmental and Biological Sciences, Universidade de Trás-os-Montes e Alto Douro, Quinta de Prados, 5000-801 Vila Real, Portugal; ltorres@utad.pt

**Keywords:** *Helicoverpa armigera*, oviposition preference, spatial pattern, Taylor’s power law, Iwao’s procedure, classification sequential plan

## Abstract

**Simple Summary:**

The tomato fruitworm, *Helicoverpa armigera*, is a key pest of several crops. It can cause particularly extensive damage in crops of processing tomatoes. Risk assessment can be a tedious and costly task if sampling protocols require a large number of plants. Sequential sampling allows sampling of a reduced number of plants when population densities are much lower or much higher than the economic or control threshold. Additionally, for crop protection purposes, sampling for classification (to assess if population density is lower or higher than the economic threshold) is adequate and requires much less effort. We studied the preferred location of eggs found on plants and then described the spatial pattern of oviposition in processing tomatoes using Taylor’s power law. Eggs were found more frequently in the exposed canopy in the upper and middle-upper strata, directly below open flower clusters, with an aggregated spatial pattern. A sequential plan was developed for 20 and 80 plants, as minimum and maximum sample sizes, respectively. This reduces sampling efforts and costs when compared to the fixed number sampling plan, and provides acceptable precision in decision-making for this pest in a processing tomato crop.

**Abstract:**

*Helicoverpa armigera* is one of the key pests affecting processing tomatoes and many other crops. A three-year study was conducted to describe the oviposition preferences of this species on determinate tomato plants (mainly the stratum, leaf, leaflet, and leaf side) and the spatial pattern of the eggs in the field, to form a sequential sampling plan. Eggs were found mainly in the exposed canopy, on *leaves a* (upper stratum) and *b* (upper-middle stratum) and significantly fewer eggs on *leaf c* (middle-lower stratum) below flower clusters. This vertical pattern in the plant was found in all phenological growth stages. The spatial pattern was found to be aggregated, with a trend towards a random pattern at lower densities. A sequential sampling plan was developed, based on Iwao’s method with the parameters of Taylor’s power law, with minimum and maximum sample size of 20 and 80 sample units (plants), respectively (two leaves/plant). For its validation, operating characteristic (OC) and average sample number (ASN) curves were calculated by means of simulation with independent data sets. The β-error was higher than desirable in the vicinity of the economic threshold, but this sampling plan is regarded as an improvement both in effort and precision, compared with the fixed sample plan, and further improvements are discussed.

## 1. Introduction

The processing tomato crop is an important industrial crop around the world, especially in China, Mediterranean countries, California, Iran, and Chile. Annually, about 37 × 10^9^ kg of processing tomatoes are produced worldwide [1]. Spain, Italy, and Portugal contribute to 90% of the European production [2]. Portugal produces 1.3–1.6 × 10^9^ kg of processing tomatoes annually [1,3], 77% for export [2], mainly in the Ribatejo region, along the Tagus River, and near the sea. Integrated production and organic farming is increasing in this region as a result of consumers and environmental demands have forced growers to change to more sustainable practices.

The tomato fruitworm, *Helicoverpa armigera* (Hbn.) (Lepidoptera: Noctuidae), is a very polyphagous and migratory species, with high reproductive potential and a propensity to develop resistance to insecticides. It is an economically important pest in many crops. Currently, it is distributed worldwide except in North America. It was recently reported in several Brazilian regions, causing billions of dollars in losses, e.g., [4]. In processing tomato crops and in Southern Europe, it is considered one of the key pests [5,6], along with eriophid mites and (since 2007) the South American tomato moth, *Tuta absoluta* (Meyrick). Although damage values are difficult to obtain, losses in tomatoes were reported to be at least 25–31.5% [7,8]. Sampling plans, both fixed and sequential, for *H. armigera* or related species such as *Helicoverpa zea* (Boddie) have been developed in different regions for soybeans, maize, cotton, and processing and fresh tomatoes, based on adult catching in pheromone traps [9], eggs [10,11,12,13,14], larvae [15,16,17,18], damage [19,20,21,22], or on a combination of these approaches [23,24].

Risk assessment methods which are based on the insect developmental stage responsible for the damage are the most appropriate, as long as they are reliable and permit the use of biological control. For tomato fruitworm, a risk assessment based either on larvae or damage does not allow for the use of *Bacillus thuringiensis* (Berl.) or baculovirus with the best efficacy, nor of egg parasitoids like trichogramma wasps [25], which are the preferred control measures in sustainable production systems. In contrast, a risk assessment based on egg counts is compatible with biological control methods and their application before the occurrence of damage. Risk assessment based on adult male catches in pheromone traps is not reliable because the correlation between the number of eggs on the plants and male catches in the traps is frequently either low or not found [26,27].

A risk assessment and decision rule model for *H. zea* was developed and successfully practiced in California (USA, Mexico) and Sinaloa (Mexico). It consisted of monitoring eggs (either parasitized or not) on 60 or 30 + 30 leaves immediately below the upper flowering cluster with open flowers (1 leaf/plant) [11,12]. Additionally, 100 green fruit were investigated, looking for larvae or recent holes. The economic threshold (ET), or control threshold, was 16 eggs excluding the parasitized ones in Sinaloa [11], or seven eggs in California [12], in 60 plants. In Australia, the ET for a processing tomato crop is five eggs per 30 leaves [13]. However, the results of these risk assessment methods and economic thresholds proved to be poor in the Ribatejo region [26] and in New Zealand [28]. Too many attacked fruits were detected with or without larvae inside them, with no previous detection of enough eggs or neonate larvae.

To develop a sampling method for risk assessment, we need to know the preferential location of the tomato fruitworm eggs and their spatial pattern. Information on within-plant distribution allows sampling to be focused on the parts of the plant where oviposition is more frequent, maintaining accuracy [29] and, consequently, reducing the time consumed in sampling and its costs. Additionally, this knowledge can also lead to the development of effective pest management strategies. The within-plant distribution of the eggs laid by *Helicoverpa* spp. in cotton is well documented [30,31,32,33]. In contrast, there is limited information about the oviposition behavior of both *H. armigera* [34,35] in other crops, and of the Plusiinae species [19,36], whose eggs (slightly flattened) can be misidentified as those of *H. armigera* (although damage caused by these species is normally restricted to leaves and is much less important). The distribution of the eggs can differ vertically in the plant between determinate processing tomato crops (which grow to a defined height, unstaked) and indeterminate crops (unlimited growth, normally staked or caged, and for fresh consumption); open field or protected tomato crops (e.g., for *H. armigera* on fresh tomato [13]; and indeterminate staked tomatoes [13,37,38], as well as on processing tomatoes mainly for *H. zea* [12,39,40]). Torres-Vila et al. [21] scouted the third leaf from the sprout terminal while testing integrated pest management (IPM) protocols, but previous tests under Portuguese field conditions [26,27] were not consistent when using this system. Since parasitism by *Trichogramma* spp. and *Telenomus* spp. may reach almost 70% in some fields [41], it is important to clarify also if these parasitoids have preferences in terms of these noctuids’ egg locations.

Field technicians’ and growers’ adherence to the sampling methods may be limited by the time involved in searching for eggs in the field. Sequential sampling can be less time consuming for some pest densities and may be a more efficient approach. For pest management decision-making, a classification decision (i.e., to evaluate if the population density is lower or higher than ET) is more useful [42,43,44,45] than estimating the population density. For classification, the effort is reduced when pest densities are either far lower or higher than the ET.

The objectives of this study were: (i) to analyze the locations of tomato fruitworm eggs in determinate processing tomatoes; (ii) to evaluate if egg parasitoids choose between these locations; (iii) to develop a sequential sampling protocol for eggs in this crop; and (iv) to evaluate its performance by simulation with independent data sets. Since Plusiinae (Lepidoptera: Noctuidae), mainly *Chrysodeixis chalcites* (Esper), *Thysanoplusia orichalcea* (Fab.) and *Autographa gamma* (L.), are other frequent noctuid species in this agroecosystem, although they have much less economic importance, their oviposition preferential sites were also studied so as to separate Heliothinae eggs from Plusiinae eggs in risk assessment procedures.

## 2. Materials and Methods

### 2.1. Study Period and Sites

This study took place in 13 processing tomato fields located in different Ribatejo locations, along the rivers Tagus and Sorraia (Table S1, Figure S1) over a 3-year period. In the first year, the study was carried out in one field (Faiel-1) to examine egg location within the field and on tomato plants. Data from seven fields observed in the 2nd and 3rd years were analyzed to study egg location on the plant, both from tomato fruitworm and from Plusiinae species. Data sets from ten of the fields were used to study the spatial pattern of tomato fruitworm eggs and to develop the sequential plans. Data from two other fields located at Lezíria Grande (Clarianos1–2 and Clarianos2–3) and data from the field observed in the first year (Faiel-1) were used for the evaluation of these plans with independent data.

Insecticide treatment of tomato fruitworm was performed according to our recommendations in most of the fields (Valada-PI, and the all Faiel, Clarianos and Coruche fields), or according to the usual practices in the region (in the others), mainly with *Bacillus thuringiensis* (Berl.), and occasionally with lambda-cyhalothrin. Treatment of tomato late blight (*Phytophthora infestans* (Mont.) de Bary) was regular in the region, mainly using the dithiocarbamates (normally mancozeb), cymoxanil and/or folpet.

### 2.2. Egg Location

In the first year, observations were made twice a week in one field (Faiel-1). At least 60 entire plants were inspected for noctuid egg position and recorded as follows. On the tomato plant (stem, leaf, flower, fruit); the leaf (leaflet, leaf side—adaxial or abaxial); the canopy (outer periphery of the plant or not exposed); plant stratum (upper, middle, lower); leaf age (young not yet developed, developed or mature, old); and orientation (N, S, W, E). The location in the field (interior or margin—first ca. 10–20 m from each side, depending on the field area) was also recorded in three more fields in the 2nd year. The plants were randomly selected in three to four field sections or systematically in a previously defined trajectory—zigzag, lines, and diagonals—alternating with each observation date. Within these trajectories, lines were randomly selected; the others were chosen by walking, distributing the 60 plants along the trajectories. This allowed a simple systematic sampling plan with a random start, which is more suitable for detecting spatial patterns than a completely random one [46,47,48], and data can be treated as if it collected at random [47,49].

The eggs were collected with a leaf portion, put in a gelatine capsule into an Eppendorf tube, and kept in the laboratory at about 25 °C ± 2 °C, 70 ± 5% relative humidity and a 14 h photoperiod, until the hatching of the larvae or the emergence of egg parasitoid adults. When larvae hatched, a first identification was made, separating Plusiinae from the non-Plusiinae (by the number of pseudopods). The larvae were reared on a maize-based artificial diet until pupation and the emerged adults were then identified.

**Figure 1:**
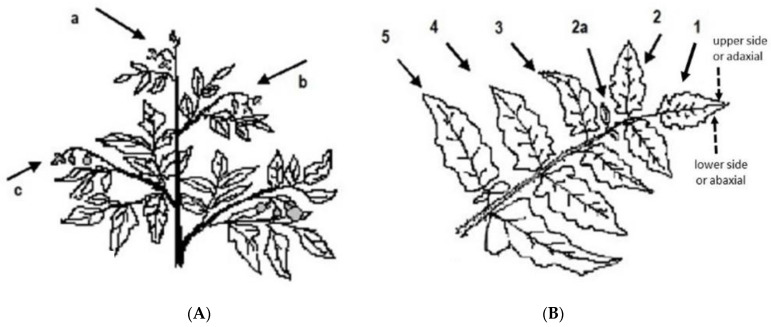
Location of eggs on plant: (**A**) position of leaves a, b, and c on the plant, and (**B**) of leaflets 1, 2, 3, 4, and 5 (the small intermediate leaflets were counted as part of the anterior fully developed leaflet, e.g., eggs on 2a were counted as on leaflet 2) [27].

After analyzing the results of the previous assay, egg monitoring was made on three leaves per plant, usually in 50–60 or 100 plants, in the 2nd and 3rd years, in seven fields (Table S1). Selected leaves were those physically immediately below flower clusters (phenologically closed or not) with well-developed flowers, open or almost open, one in each vertical stratum of the plant—one in the upper stratum (*leaf a*), one in the upper-middle stratum (*leaf b*), and the third in the middle-lower stratum (*leaf c*) (Figure 1a). All leaves were chosen in the top or laterally in the exposed canopy and plants were selected as mentioned above, but the first plant in each line was defined, randomly, after walking 10–20 m to avoid the field margin. The location of the eggs was recorded (leaf, leaflet, and leaf side) (Figure 1b). The eggs were collected and treated as described above.

Field observers distinguished correctly 97.4% of the eggs from which *H. armigera* larvae emerged and 90.5% of the eggs from which Plusiinae larvae emerged (as the later eggs are a little more flattened). Therefore, data from the eggs (damaged, unviable, and parasitized) that had been identified in the field as tomato fruitworm or Plusinae were also used in the analysis. As the egg parasitism rate had reached 70% in some fields [41], not using these eggs could lead to bias in the location study if egg parasitoids preferred certain locations or certain noctuid hosts to oviposit.

### 2.3. Spatial Pattern and Sequential Plans

The stratified systematic sampling specified above for studying egg location was performed, weekly or twice a week, in year 2 and 3 in 10 tomato fields (Table S1), observing 50 to 105 plants, except for Coruche fields where 30 plants were observed (exceptionally, one data set had only 17 plants). Only eggs located on leaf a and leaf b were used in this analysis, since the location study concluded that these leaves were preferred for fruitworm oviposition. *Spodoptera* spp., *Peridroma saucia* (Hbn.), and sometimes *A. gamma* but never *H. armigera,* lay their eggs in clusters. Thus, all the eggs that were laid in clusters were not considered. The spatial pattern was studied to achieve a practical sequential sampling plan, and it is believed that growers do not easily distinguish between tomato fruitworm and Plusiinae species eggs. Moreover, as the population density of Plusiinae is in general much lower than the population density of *H. armigera,* and the economic importance of the tomato fruitworm attacks are much higher, the risk associated with this option is eventually to carry out one unnecessary treatment. After examining the results obtained in the egg location study, all the eggs located on leaf a and leaf b were used for the remainder of the study.

### 2.4. Data Analysis

#### 2.4.1. Egg Location

For the egg location study, we examined the eggs from which larvae emerged and the eggs identified in the field that were unviable, damaged, or parasitized, according to each of three main phenological stages of the tomato crop—flowering, green fruit, and maturing-senescence—using nonparametric Friedman and Wilcoxon-matched pair tests. To compare the location of tomato fruitworm and Plusiinae eggs, we used the proportion of eggs found in each location in relation to the total number of eggs of that taxa in each observation date and field, since the population densities of *H. armigera* and Plusinae are quite different. The same was done to compare the location of parasitized and not parasitized eggs. The study concerning egg location in the field was performed by comparing the number of eggs per plant located in the interior of the field and the field margin, from the fields and observation dates where these data were recorded, using the Wilcoxon matched-pair test. These tests were performed with IBM SPSS v24 [50].

#### 2.4.2. Spatial Pattern and Sequential Plan

The spatial pattern of tomato fruitworm was described using Taylor’s power law (TPL) to model the variance–mean relation of the sample: ${\hat{s}}^{2}={a\bar{x}}^{b}$, where $\bar{x}$ and ${\hat{s}}^{2}$ are the sample variance and the sample mean, respectively, and *a* and *b* are the parameters of TPL. The parameter *a* is a scaling factor, dependent on the habitat and sampling method, and the exponent *b* is a measure of aggregation. TPL parameters were estimated (i.e., *a* and *b*) by regressing $log_{10}s^{2}$ against $log_{10}\bar{x}$ with a type II regression model, since $\bar{x}$ was also estimated. For this purpose, a reduced major axis regression method was used, utilizing an add-in for an Excel^®^ datasheet [51] after verifying the assumptions. The observations in which no eggs were recorded, or those in which only one egg was observed on only one plant (for the model, four and six data sets, respectively; for validation, one and three data sets respectively, and two data sets excluded due to the low number of observed plants) were excluded from all analyses, since they would lead to pseudo-randomness as a result of mean–variance duplicate combinations [52].

Various data sets (corresponding to observations made on different sampling dates) from each field were used, as in other studies of *H. armigera* or *H. zea*, e.g., [13,16,17,37] or other pests, e.g., [47,53,54]. Tomato fruitworm has at least three generations in the Ribatejo region and is a strong-flying insect, highly migratory, and highly polyphagous; therefore, we assumed the data sets to be independent, and regression for estimation of the TPL parameters could be carried out. Moreover, type II regression is more robust to the lack of independence [55]. However, graphical analysis of the dispersal residuals through time was made for each field to verify if there was any tendency of dependence. TPL parameters were also estimated by year and by zone to verify if the spatial pattern of the tomato fruitworm remained consistent over time and across locations. Confidence intervals for the TPL *b* parameter and comparison between *b* values for each year and zone were compared using F tests of homogeneity of the regression slopes [56] using (S)MATR 1.0 [57].

Some authors have argued that the negative binomial distribution (NB), being a probability model, should be the preferred method used to describe a population and on which to base a sampling plan [58]. We thus first tried to compute the k parameter of the NB by the maximum likelihood method for data from each observation date and field, and we examined the fitness of the sample data to the NB by the calculation of the T or U statistics, since the number of frequency classes was too low to achieve sufficient sensitivity in the Chi-squared goodness of fit test [48,59]. Moreover, Iwao’s patchiness regression method was also attempted.

To develop sequential sampling plans, Iwao’s, Wald’s, and the converging lines sequential methods were attempted. The variance used to calculate the stop boundaries was based on the TPL parameters from the model of the spatial pattern estimated above, calculating the dispersion parameter (k) of the NB from k = μ^2^/(σ^2^ − μ). The economic threshold (critical density) was empirically determined by Figueiredo et al. [27] as 8–9 eggs on 60 plants (0.13 to 0.15 egg/plant), considering leaf a and leaf b. Acceptance curves (stop boundaries) were calculated using the Crop Protection Decision-Making Mathcad 8.0 worksheets from the electronic version of Binns et al. [45] with an ET of 0.15. The performance of the sampling plans was determined by estimating the operating characteristic (OC) curve, which is the probability of not rejecting H_0_: μ < ET, and the average sample number (ASN) curve, which represents the average number of sample units required to stop sampling, by means of a simulation using the TPL validation regression’s parameters (based on data sets independent from the model). These curves should be used in order to judge the performance of sample plans in pest management decision-making, e.g., [42,44,45], and operating characteristics (OC) and average sample number (ASN) functions were estimated by simulation using the same mcadcpdm worksheets, but with the TPL regression performed with the 34 data sets from the validation fields (Table S1). These last data sets were not used for the estimation of the model’s TPL parameters. The ranges of means used for the model and for the validation of TPL regressions were found to be similar, and the number of means above and below the ET was approximately the same in both TPL regressions.

For the estimation sampling plan, the minimum sample size (n) and the stopping line (T_n_) were calculated as:

$$n={a\bar{x}}^{\left(b-2\right)}/D^{2}$$

and

$$T_{n}=\;{\left(D^{2}/a\right)}^{\frac{1}{b-2}}{\;n}^{\left(b-1\right)/\left(b-2\right)}$$

where D is the precision level and *a* and *b* are the parameters of TPL regression [48,60].

The validation of this last sampling plan was carried out using re-sampling analysis with the same 34 independent data sets over 1000 iterations using the TPL parameters of the model, the software RVSP2 (Resampling for Validation of Sample Plans) [61], and the precision level recommended for ecological density estimation (0.10–0.15) [48]. The mean level of precision obtained after 1000 iterations of each data set was compared with the nominal precision level, which was then eventually adjusted. The number of samples needed to satisfy the precision level required as a function of the sample mean was plotted.

For comparison with the classification sequential plan, a precision level of 0.30 was also used, since a level of 0.25–0.30 is acceptable for pest management purposes [48] and has been used worldwide, e.g., [13,54,62,63].

## 3. Results

### 3.1. Egg Location

Almost all the eggs were *H. armigera* and Plusiinae; very rarely eggs of *Heliothis peltigera* (Den. and Schiff.), *P. saucia*, or *Spodoptera exigua* (Hbn.) were found. *H. armigera* was by far the most abundant species in all the fields and years (≈80%). The Plusiinae species identified after adult emergence were *T. orichalcea* (60.4% of the Plusiinae), *A. gamma* (28.3%), *C. chalcites* (10.9%), and *Trichoplusia ni* (Hbn.) (0.4%). Only those from *H. armigera* and Plusiinae (all species together) were analyzed.

The number of eggs laid per plant was statistically higher in the margins than the interior of the field in one (Canha-3) of the four fields where this position was recorded (Wilcoxon matched pair test: Z = −2.272, *p* = 0.023, N = 13). Globally, considering data from the four fields all together, statistical differences were found only at a 90% confidence level. The correlation between eggs per plant in the margins and the interior of the field was high and statistically significant (Spearman’s rho coefficient = 0.714, *p* < 0.001, N = 43). In the first year, in Faiel-1, almost all tomato fruitworm and Plusiinae eggs were found on the plants’ exposed canopies. This fact was the basis of the selection of the three leaves to be used in the observation method for the remainder of the study. No differential behavior was found regarding the orientation.

#### 3.1.1. Tomato Fruitworm Eggs

A total of 1012 eggs identified as eggs of *H. armigera* (parasitized or not) were used in the analysis. In the nine study fields, 40% of tomato fruitworm eggs, on average, were found on leaf a, and another 40% on average on leaf b (Table 1). Significantly fewer eggs were detected on leaf c (Friedman test: χ^2^ = 21.44, *p* < 0.001, N = 87) (on average about 20%) (Table 2). Only 0.8% of the eggs were found on the stems, flowers, and fruits. Most *H. armigera* eggs were laid on the most terminal leaflets—on average, 94.1% of the eggs were detected on the three first leaflets and almost no eggs were found on leaflet 5 (Table 1). On any of the three terminal leaflets more eggs were laid than on leaflet 4 or 5 (Friedman test: χ^2^ = 153.49, *p* < 0.001, N = 87) (Table 2). With regard to the surface of the leaf, tomato fruitworm females laid significantly more eggs on the lower (abaxial) side of the leaf (Friedman test: χ^2^ = 50.24, *p* < 0.001, N = 87) (Table 2). The eggs laid on the lower side of the leaf represented 69% of the total number of eggs, and the mean proportion per observation date was 79%.

The preference for oviposition on leaves a and b was consistent throughout the phenological crop growth (Table 3), although in the “green fruit” stage the differences were not statistically significant. Only in the case of the “flowering” and “green fruit” stages combined did the more conservative comparison of medians reveal that leaf a and leaf b were both significantly different from leaf c at a 95% confidence level.

#### 3.1.2. Plusiinae Eggs

A total of 300 eggs were identified as eggs of Plusiinae (parasitized or not) and were used in the analysis. The number of Plusiinae eggs was significantly higher on leaves b and c than on leaf a (Friedman test: χ^2^ = 18.28, *p* < 0.001, N = 69) (respectively ≈40–45% and less than 20% of the eggs, by observation date), and on leaflets 1, 2, and 3 than on leaflets 4 or 5 (on which almost none were oviposited) (Friedman test: χ^2^ = 102.17, *p* < 0.001, N = 69), and on the lower side of the leaves (Friedman test: χ^2^ = 40.32, *p* < 0.001, N = 69) (Table 1 and Table 2).

#### 3.1.3. Egg Location—Comparison between *Helicoverpa armigera* and Plusiinae

On leaf a, the proportion of tomato fruitworm eggs was significantly higher than the proportion of Plusiinae eggs (Table 1). The inverse was found on leaf c, where a significantly greater proportion of eggs of Plusiinae were found. Moreover, significantly more eggs of *H. armigera* than from Plusiinae were found on leaflet 4 and on the upper (adaxial) side of the leaf (Table 1).

#### 3.1.4. Egg Location—Comparison between Parasitized vs. Non-Parasitized Eggs

A higher proportion of parasitized eggs than of non-parasitized eggs of both Heliothinae and Plusiinae were found on leaflet 1 and fewer on leaflet 3 (Table 1). No significant differences were found in relation to the location on the plant (leaf, leaflet, and leaf side) or in the field (margin vs. interior). Note that field location (interior, margin) was recorded in only four of the fields.

### 3.2. Spatial Pattern in the Field

Although most of the data sets fitted the NB distribution, it was not possible to get a common k, since it varied with the density of fruitworm eggs. Moreover, Iwao’s patchiness regression, which usually fits better than TPL [52], did not provide a consistent fit (r^2^ = 0.13). Furthermore, TPL residuals through time did not reveal any trend of dependence amongst the data.

**Figure 2:**
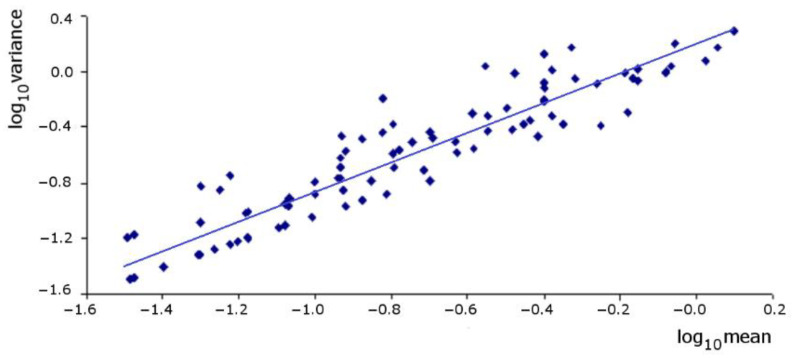
Taylor’s power law (TPL) regression plot for *Helicoverpa armigera* in processing tomato crops (the regression model was fitted by a reduced major axis regression method with 105 data sets; the line corresponds to the trend line).

TPL described the variance–mean relationship well (r^2^ = 0.879, F = 751.10, *p* < 0.0001) (Figure 2, Table 4). The slope parameter was significantly higher than unity (F = 14.25, *p* = 0.0003), although just slightly, and the intercept was also higher than 1, indicating an aggregated within-field spatial pattern with a trend towards a random pattern at lower densities. TPL also described well the variance–mean relation per year and per location (r^2^ between 0.814 and 0.955; *p* < 0.0001 in all cases); the intercept and the slope were always higher than the unity (*p* < 0.05 in all cases), and the 99% confidence interval for the slope always contained the value of the model’s slope (the same happened with the 95% confidence interval, excluding the Valada location) (Table 4). Comparing the *b* values from different locations and years, significant differences were not found at a 99% confidence level, thus implying no significant year or location effects. However, the values of the parameter *a* of TPL were much more variable across locations and years (Table 4).

### 3.3. Sequential Plans

#### 3.3.1. Sequential Plan for Tomato Fruitworm Egg Density Classification and Its Validation

**Figure 3:**
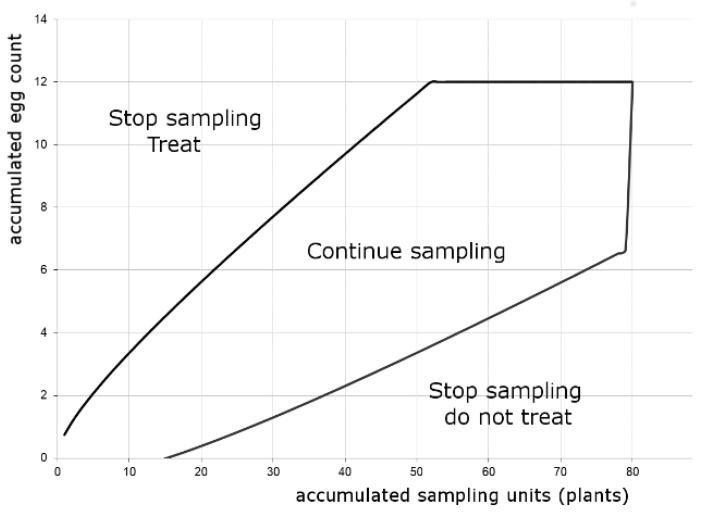
Acceptance curves (stop boundaries) of the sequential sampling plan for *Helicoverpa armigera* eggs according to Iwao’s method (based on the TPL regression with 105 data sets—model values in Table 4; α = 0.20, β = 0.10; n_min_ = 20, n_max_ = 80, σ = 0.16); the boundaries were calculated using crop protection decision-making Mathcad worksheets of the electronic version of Binns et al. [45].

Several sequential sampling plans for risk assessment purposes were developed using the TPL parameters from the model and by Wald’s, Iwao’s, and converging lines methods. Considering the OC curves, the sampling plans developed with Iwao’s method performed best. Increasing the maximum sample number from 60 to 80 plants did improve the OC and, on average, did not force a substantial additional effort (Figure S2). Increasing this maximum to 100 plants did not improve the OC curve and increasing to more than 100 plants would make the sampling plan infeasible (the necessary simulations were performed). Increasing the minimum sample number from 20 to 30 did not improve the plan either. Decreasing the α-error slightly improved the OC curve but not enough to consider the higher sampling effort. Therefore, the sequential sampling plan was developed considering a minimum and a maximum sample number of 20 and 80 plants, respectively, α-error = 0.20 and β-error = 0.10. The stop boundaries of this sequential sampling plan are presented in Figure 3. In each plant, one leaf a and one leaf b should be observed. OC and ASN curves are presented in Figure S2a,b. Only simulations with a mean density very close to ET stopped at the maximum sample size of 80 plants; even the ASN 90% percentile curve reached 80 plants for a very limited range of egg densities (Figure S2c).

#### 3.3.2. Sequential Plan for Tomato Fruitworm Egg Density Estimation and Its Validation

A sequential plan (stop lines) for estimation of tomato fruitworm egg density for ecological studies was also determined, and the mean, minimum, and maximum sample sizes needed to achieve an intensive sampling fixed precision were determined by bootstrap simulation with RVSP2 (Figure S3). A precision of 0.15 was used (requesting a precision of 0.14 by simulation), since at 0.10 the validation was not possible even with replacement for the five data sets with lower densities (less than 0.08 eggs/plant). The re-sampling analysis to achieve a precision of 0.15 was run and all data sets were successfully processed excluding the lower density one ($\bar{x}=0.02$) which ran with difficulty, not achieving the 1000 iterations. The sample size calculated theoretically for 0.02 eggs/plant and 0.03 eggs/plant was 2702 plants and 1904 plants, respectively; the bootstrap estimated mean sample size for a density of 0.03 eggs/plant was 1389 plants.

## 4. Discussion

The great majority of the eggs (≈80%) found on the studied fields were from *H. armigera*. Both tomato fruitworm and Plusiinae species revealed a tendency towards higher oviposition in the margins of the fields. However, this fact alerts us to a possible overestimation of pest density if risk assessment includes the plants in the field margins, which are commonly recommended to be avoided [49].

Regarding the location in the plant, almost all the eggs of *H. armigera* were found to be laid on the exposed canopy in the first year of the study. Therefore, we decided to observe three leaves at different heights in the external part of the plant in the following years. These results are in accordance with other authors who found that most of the eggs of *H. zea* were laid on the periphery of the determinate tomato plant [64,65]. We found almost no eggs on plant organs other than leaves. This preference was found in other studies of *H. armigera* [28] and *H. zea* [39,64,65], where very few eggs were laid on blooms and fruits, and none on stems. In contrast, Zitsanza et al. [66] reported an important proportion of eggs on flowers, and Saour and Causse [67] found about 13% of the eggs on flowers, fruits, and stems in an indeterminate tomato plant. Regarding the stratum where the external leaf is located, we found a clear preference of the tomato fruitworm to oviposit on leaves positioned in the upper and upper-lateral part of the canopy. Zalom et al. [12] also found this result in California for *H. zea*, with an identical proportion of eggs, but Snodderly and Lambdin [40] found more *H. zea* eggs in the middle plant strata in fresh tomatoes. On indeterminate tomatoes, most of the *H. armigera* eggs were laid on the upper half, between the highest cluster (not yet open) and the third cluster (with very recent fruits), more or less equivalent to leaves a and b on a determinate plant [67]. Moreover, we did not find differences in preference to oviposit throughout the tomato phenological cycle, contrary to Zitsanza et al. [66] and Braswell et al. [33], for *H. armigera* on tomato, and *H. zea* in cotton, respectively.

Both taxa preferred the three terminal leaflets and the lower surface of the leaf. However, we found that when the weather is cloudy and cooler more eggs are seen on the upper side of the leaves (data not shown). The preference of *H. armigera*/*H. zea* for the lower leaf side corroborates the findings of other authors, e.g., [40,64,68], and contradicts some other studies, e.g., [39,67]. Araújo [68] had already detected the preference for the terminal leaflets. The differences reported here are not strange, since geographic differences in oviposition preference sites have already been noticed in Heliothinae species [69].

The Plusiinae preference to oviposit in tomato plant strata lower than those used by the tomato fruitworm was already verified before for *H. armigera* [36] and for *H. zea* related to *T. ni*. The latter was found to have proportionally fewer eggs laid below the higher flower cluster [12], as we observed with the Plusiinae species present in the fields of the Ribatejo region.

No difference was detected among the leaf location related to parasitism. Since *Trichogramma* and *Telenomus* species found in this region have no preference between these noctuid species [41], this analysis was not biased by host insect preference. Therefore, our results suggest that egg parasitoids have no preference site on which to lay their eggs and that all such noctuid eggs have the same chance to be parasitized wherever they are located, which is expected, since these species are generalist parasitoids [68].

In conclusion, for risk assessment purposes, the sampling effort can be reduced by searching for eggs only on leaf a and leaf b. Moreover, searching for noctuid eggs on leaf a and leaf b contributes to avoiding overestimating tomato fruitworm eggs by taking Plusiinae eggs into account, due to their differential preference for *leaf c*. Although more tomato fruitworm eggs were found on the lower leaf side, it is recommended to look for eggs on both sides for risk assessment, since it requires little further effort and allows for the detection of 20–30% more of the eggs. Regarding egg location within the tomato plant, for tomato fruitworm risk assessment purposes, we recommend scouting for eggs on the two sides of the leaf of the first three leaflets of the two leaves physically below clusters with open or almost open flowers in the exterior of the canopy in upper and middle-upper strata (leaf a and leaf b).

It is common to fail in finding a common k even when data fit the negative binomial distribution [42,48,52], and the good fit to TPL here achieved is in accordance with this difficulty of finding a common k [70]. Some authors note that sample means extending down to low densities tend to decrease the slope parameter (*b*) and to increase the intercept (log *a*). Perry and Woiwod [71] defined the inferior limit for these low density means as $m=a^{\left(1/\left(1-b\right)\right)}$, which in our case would be *m* = 0.013 (for TPL model) and *m* = 0.014 (for validation TPL). The inferior limit of our means’ ranges was higher than these values (Table 4) and so there was no risk that slopes were decreased by this fact. The *b* values obtained in this study are in accordance with the values found in the literature for *H. armigera* and *H. zea* in tomato and other crops—1.23 for *H. armigera* eggs [37] and 1.45–1.62 for *H. armigera* and *H. punctigera* (Wall.) eggs [13], both in indeterminate tomatoes; 1.08–1.11 [72], 1.13–1.14 [73], 1.28–1.34 [14], and 1.18 or 1.31 [74] for *H. zea* eggs (parasitized or not, separately or all together with *H. punctigera*) in processing tomatoes, cotton, soybeans, and sorghum, respectively; and 0.926 for *H. zea* larvae in sweet corn [16]. The variability among *a* values is common due to its variation with environmental conditions but they were also similar to those reported by these authors, which ranged from 0.84 to 6.45, with values around 1.8–2.2 being more commonly found.

Our results show that, for densities sufficiently lower or higher than the ET, it is enough to sample less than the 60 plants recommended in the fixed number sampling plan presently used by field technicians for risk assessment, while still maintaining precision. Consequently, the sequential sampling presented here provides a tool for reducing sampling efforts on several occasions. Simultaneously, it shows the likelihood of performing unnecessary treatments when densities are lower than the ET, or of not performing treatment when densities are higher than the ET (α and β errors, respectively), which is especially important for densities near the ET, providing an estimate of the error associated with the decision-making process.

The OC curves demonstrate that the probability of wrong decisions is very low when the egg density is either well below or well above the ET. Nevertheless, the OC should be steeper than was obtained here to provide a better precision in the 30% vicinity of the ET, which is the value usually referred to in the literature as the acceptable limit of error probability higher than the chosen α and β (e.g., [42]). The error obtained by simulation was much higher than desirable (Figure S2a), especially the β-error, which was near 20% for a mean number of eggs/plant around 0.20, indicating a higher risk level of considering the egg density lower than ET when this is not true and when control action should in fact be taken. This problem can be minimized if sampling periodicity is shortened from one week to half a week when sampling results conducted in relation to a non-treatment decision with densities near ET. Inversely, a very low egg density could lead to a larger interval for the next sampling, if a time dimension is included in the sampling plan for adjusting the time interval between two consecutive samplings [45,58]. This would decrease risk assessment costs. The attitudes of growers facing risks and the demands of the destination market also influence how to deal with this uncertainty near ET.

The difficulty in harmonizing reliability and desirable precision of the sample mean estimates and sampling plan feasibility, as well as the cost of the estimation (including time spent on this operation), is a very common problem. For example, Ho [47] was forced to accept a sampling plan with a D = 0.8 since higher precision estimates would lead to an impracticable sample size. Performance of sampling plans developed to control the spotted tentiform leafminer in apple orchards analyzed by simulation also revealed high β-errors in the 30% vicinity of the ET; however, despite that, Nyrop et al. [75] found that only on one occasion was a non-treatment decision was taken wrongly when the sampling plan was evaluated in the field, and concluded that its use would result in significant reductions of insecticide applications. Cullen et al. [76] also reported this conflict between precision and feasibility, recommending a sampling plan with an 85% treatment decision certainty and a precision range of 0.43 for the consperse stink bug in processing tomato commercial fields in California.

The improvement of this plan requires taking into account the high egg parasitism level, sometimes around 50% or higher, by *Trichogramma* spp. and *Telenomus* sp., in the Ribatejo processing tomato fields [41]. The ET value was assessed considering that part of the eggs would be parasitized, but this parasitism rate is not similar amongst fields across years and during the entire season. Taking into account the real parasitism rate in each field and observation date would be a better approach. The same approach was used for *H. zea* in California [72,77]. Since sampling procedures and control interventions increase crop costs, they both have to be minimized [43], so conservation biological control has to be incremented by adopting some cultural practices in the field (such as allowing the presence of ecological infrastructures) and taking this parasitism into account in crop protection decision-making.

An improvement in ET estimation would also reduce decision errors. The actual value of ET was estimated by empirical studies. Crop loss assessment studies would improve knowledge of the ET and indirectly improve any sampling plans [44,45].

Zalom et al. [19] reported 1.56 min/plant as the time needed for picking up eight leaves from a plant and counting the eggs on them; we found about 1.25 min/plant to be necessary for observing one leaf/plant, including in this value the time to move between plants to sample a 4–5 ha field, when sampling is done early in the morning. Considering two leaves per plant and 60 plants/sample, sampling *H. armigera* eggs would last about one hour, which is the time proposed for sampling tomato fruitworm larvae on tomatoes in New Zealand [28] and the maximum for sampling eggs and larvae in Spain [78]. The cost of these risk assessment methods can be a difficulty in the adoption of IPM/integrated production by the growers, even considering that they would be executed only during the sampling period. This period begins with the increase in male captures in pheromone traps, at or after flowering, and ends when the harvestable fruits are mature (some fruits will be too green to be collected when the harvest is done and so protecting them is economically loss-making). In this study, time spent in sampling was associated with the direct observation of the leaves in the plant. Picking up the leaves and observing all together afterwards would lead to a more rapid and less fastidious sampling procedure. Moreover, the extrapolation of a field’s sampling results to the others in its vicinity with the same phenology (the same cultivar and plantation date, homogeneous soil conditions, and identical cultural practices) would lead to a reduction the time spent on the sampling procedure and its costs, but the limits of this extension and the risks associated should be investigated.

Another approach to minimizing the sampling costs is to develop time-sequential sampling for classification, which would lead to changing time intervals according to pest densities observed in the field [45].

Nevertheless, the sequential sampling plan obtained and validated in this study can be adapted to other processing tomato production regions, namely those with a Mediterranean climate around the world, improving the sustainability of these production systems.

In a sequential sampling plan for the estimation of *H. armigera* egg density, using the same TPL regression for the model and the same validation fields, the average sample size needed to satisfy the stopping rule estimated by bootstrap simulation was 129 plants (ranging from the minimum sample size set at 20 plants for a density of 1.26 eggs/plant to a maximum of 1342 plants for a density of 0.02 eggs/plant) to achieve the inferior limit of precision generally acceptable for crop protection purposes (D = 0.30) [48,60]. These sample sizes are clearly higher than in the above mentioned sequential sampling plan developed for classification and are higher than is achievable in the crop field for risk assessment purposes.

## 5. Conclusions

Almost 80% of tomato fruitworm eggs were laid in the upper and upper-middle strata in the periphery of the canopy of the tomato plant. Therefore, two leaves were selected immediately below flower clusters (one in the upper and one in the upper-middle stratum) for use in the sampling plan. The tomato fruitworm was observed to have a contagious or aggregated spatial pattern, with a trend towards a random pattern at lower population densities. We developed and validated a classification sequential sampling plan which is adequate for risk assessment purposes in crop protection. It needs a minimum and a maximum of 20 and 80 sampling units (plants), respectively, observing two leaves in the upper and upper-middle strata in each plant. This plan is an improvement on the current 60-plants fixed sampling plan, by reducing effort and associated costs on several occasions, since it can be less time consuming, with an acceptable risk in the decision-making process, and can be adapted to other processing tomato production regions.

## Figures and Tables

**Table 1:** Location of *Helicoverpa armigera* and Plusiinae eggs in processing tomato crops in Ribatejo—proportion (in %) of tomato fruitworm eggs and of Plusiinae eggs on each leaf, leaflet, and leaf side ^1^, and mean proportion of parasitized and non-parasitized eggs of tomato fruitworm and Plusiinae (SE-Standard error of the mean).

Plant Part	Position	Mean Proportion of Eggs of (±SE) (%) ^2^		Parasitism—Mean Proportion of Eggs (±SE) (%) ^3^	
		*H. armigera*	Plusiinae	Non-Parasitized	Parasitized
Leaf	a	39.4 ± 3.3 a	16.9 ± 3.1 b	38.3± 3.2 A	28.7± 3.0 A
	b	40.3 ± 3.1 a	45.0 ± 4.2 a	39.7± 3.4 A	40.1 ± 2.9 A
	c	19.5 ± 2.4 a	38.2 ± 4.3 b	26.4 ± 3.0 A	31.2 ± 3.4 A
Leaflet	1	33.6 ± 3.2 a	42.7 ± 4.7 a	33.2 ± 3.0 A	42.5 ± 3.3 B
	2	36.1 ± 3.2 a	30.7 ± 4.2 a	33.1 ± 3.0 A	38.5 ± 4.1 A
	3	24.4 ± 1.6 a	24.8 ± 4.0 a	26.5 ± 2.8 A	16.5 ± 2.4 B
	4	6.7 ± 1.6 a	1.8 ± 0.9 b	6.9 ± 1.8 A ^5^	2.4 ± 0.8 A ^5^
	5	0.2 ± 0.1 a	0.0 ± 0.0 a	0.3 ± 0.3 A	0.3 ± 0.2 A
Leaf side	lower	79.0 ± 2.4 a	86.0 ± 3.4 b	81.8 ± 2.9 A ^4^	75.1 ± 3.7 A ^4^
	upper	21.0 ± 2.4 a	14.0 ± 3.4 b	24.6 ± 2.9 A ^4^	15.7 ± 2.6 A ^4^

^1^ Mean values are related to 87 (tomato fruitworm) or to 69 (Plusiinae: leaf and leaflet and leaf side, respectively) observation dates in which eggs were found, in seven processing tomato fields (Faiel-2, Faiel-3, Foz-3, Valada-PI2-3, Valada-LQ-3, Valada-AB-3, and Canha-3); 1012 eggs of tomato fruitworm and 300 eggs of Plusinae species of which the identification was achieved. **^2^** Of the total number of eggs of those taxa detected in all leaves and leaflet positions or both leaf sides, respectively, on each observation date; for each plant part and position (within the same line); mean values followed by a different letter correspond to medians that are statistically different (*p* < 0.05) (Wilcoxon matched pair test—N = 58 (leaflet); N = 59 (leaf, leaf side)). ^3^ Proportion (%) of parasitized and non-parasitized eggs on each leaf, leaflet, and leaf side in relation to the total number of parasitized or non-parasitized eggs, respectively, detected in all leaves and leaflet positions or both leaf sides, respectively, in each observation date. Mean values are related to 87 (non-parasitized) or 75–76 (parasitized, leaf and leaflet, leaf side, respectively) observations in which eggs and parasitized eggs were found, in the same seven processing tomato fields; for each plant part and position (within the same line); values followed by a different letter correspond to medians that are statistically different (*p* < 0.05) (Wilcoxon matched pair test—N = 73 (leaf, leaflet) or 74 (leaf side)). ^4^ The sign test was performed since the assumptions of the Wilcoxon matched-pair test were not met (N = 73 (leaflet) or 74 (leaf side)).

**Table 2:** *Helicoverpa armigera* and Plusiinae eggs in processing tomato crops in Ribatejo ^1^—comparison among the locations within leaf, leaflet, and leaf surface for tomato fruitworm and Plusiinae ^2^ (SE—Standard error of the mean).

Plant Part	Location	Egg Mean Number per Observation Date (±SE) ^2^	
		*Helicoverpa armigera*	Plusiinae
Leaf	a	4.16 ± 0.65 *a*	0.80 ± 0.18 A
	b	4.37 ± 0.58 *a*	1.90 ± 0.28 B
	c	2.46 ± 0.41 *b*	1.38 ± 0.24 B
Leaflet	1	4.16 ± 0.61 *a*	1.68 ± 0.26 A
	2	3.69 ± 0.57 *a*	1.30 ± 0.23 A
	3	2.63 ± 0.40 a	0.87 ± 0.13 A
	4	0.70 ± 0.13 *b*	0.09 ± 0.03 B
	5	0.06 ± 0.04 *b*	0.00 ± 0.00 B
Leaf side	lower	7.77 ± 0.92 *a*	3.77 ± 0.57 A
	upper	3.48 ± 0.67 *b*	0.45 ± 0.10 B

^1^ Mean values are related to 87 observations (tomato fruitworm) or to 69 (Plusiinae) observation dates in which eggs were found, in seven processing tomato fields (Faiel-2, Faiel-3, Foz-3, Valada-PI2-3, Valada-LQ-3, Valada-AB-3, and Canha-3); 1012 eggs of tomato fruitworm and 300 eggs of Plusinae species of which the identification was achieved were used. ^2^ Medians corresponding to each plant part and each taxonomic group (within the same column) of which the mean values are followed by a different letter are statistically different (*p* < 0.05) by multiple comparison of medians tests after the Friedman test (Friedman test: *p* < 0.001, except comparisons *H. armigera leaf a–leaf c* with *p* = 0.005 and Plusiinae *leaf a–leaf c* with *p* = 0.032).

**Table 3:** Location of *Helicoverpa armigera* eggs within the plant in processing tomato crops in the Ribatejo region—comparison of the number of eggs in each location per phenological stage—results from Friedman nonparametric tests and multiple comparison of the medians ^1^ (SE—Standard error of the mean).

Phenological Stage	Mean ± SE			Friedman Test		
	a	b	c	N	χ^2^	*p*
Flowering	4.96 ± 1.44 ab	4.78 ± 1.02 a	2.28 ± 0.64 b	28	12.9	0.002
Green fruit	3.70 ± 0.98 a	4.44 ± 1.26 a	2.59 ± 0.87 a	27	3.34	0.188
Flowering + green fruit	4.34 ± 0.87 a	4.61 ± 0.80 a	2.43 ± 0.57 b	55	14.9	0.001
Maturing-senescence	3.97 ± 0.95 a	4.06 ± 0.78 a	2.58 ± 0.68 a	31	7.02	0.03

^1^ Values related to 86 observations (one observation was not considered because it was related to a “before flowering” phenological stage) in which *H. armigera* eggs were found, in seven processing tomato fields (Faiel-2, Faiel-3, Foz-3, Valada-PI2-3, Valada-LQ-3, Valada-AB-3, and Canha-3), and to a total number of 1012 eggs of *H. armigera* of which the specific identification was achieved. Medians corresponding to phenological stages of which the mean values (in each line) are followed by a different letter are statistically different (*p* < 0.05).

**Table 4:** Estimates of the parameters of Taylor’s power law (slope and intercept) for *Helicoverpa armigera* in processing tomato crops and 95% and 99% confidence intervals for the slope fitted to the data sets of the model, validation fields, and each location and year (N—sample size, SE—standard error of the mean).

TPL	N	Range Means	*b* ^(1)^	SE *b*	*a*	Confidence Interval for *b*				r^2 (2)^
						95%		99%		
Model	105	0.03–1.26	1.137 ***	0.039	1.810	1.063	1.217	1.04	1.244	0.879
Leziria Grande	72	0.02–1.26	1.107 *	0.047	1.696	1.017	1.206	0.989	1.24	0.871
Valada do Ribatejo	43	0.03–0.48	1.339 ***	0.077	3.184	1.180	1.498	1.126	1.552	0.858
Canha + Coruche	24	0.03–0.88	1.279 *	0.112	2.630	1.058	1.546	0.990	1.653	0.814
2002	11	0.02–0.47	1.196 *	0.077	2.118	1.019	1.403	0.952	1.503	0.955
2003	49	0.05–1.26	1.161 *	0.070	1.887	1.026	1.314	0.985	1.369	0.821
2004	79	0.03–1.26	1.164 ***	0.048	1.977	1.072	1.262	1.044	1.296	0.871
Validation fields	34	0.02–1.26	1.181 **	0.063	2.155	1.056	1.321	1.016	1.373	0.902

^(1^^)^ Slope significantly different from 1.0 in all cases, with the following significance level (*p*) for the F statistic, calculated by (S)MATR [57]: *p* < 0.05 (*), *p* < 0.01 (**) or *p* < 0.001 (***). ^(2)^ Significance level of the regression was lower than 10^−7^ in all cases.

## Data Availability

The datasets used and/or analyzed during the current study are available from the corresponding author on reasonable request.

## Supplementary Materials

The following are available online at https://www.mdpi.com/2075-4450/12/1/13/s1, Figure S1: Location of the monitored fields in the Ribatejo region (Portugal); Figure S2: Sequential sampling for risk assessment: OC (a), ASN (b), and ASN quartiles/ percentiles; Figure S3: Sequential sampling for density estimation in ecological studies; Table S1: Main features of the processing tomato fields monitored and data obtained; Table S2: Comparison of the number of eggs per plant in the margin and the main interior of tomato fields.
