# Supplementary material for: Risk Assessment for Tomato Fruitworm in Processing Tomato Crop-Egg Location and Sequential Sampling
Source: Insects. 2020 Dec 28;12(1):13. doi: 10.3390/insects12010013 (PMC7824523; doi:10.3390/insects12010013)
Supplement: Supplementary file 1 [file insects-12-00013-s001.pdf]

Table S1. Main features of the processing tomato fields monitored and data obtained.

Field (locations)[1]	Year	Area (ha)	Soil[2]	Plantation date	observation dates (N)	observed plants (total) (N)	sampled eggs (total) (N)	Studies where data was used[3]
Faiel-1 (LG)	1	5.0	H	2 May	15	1132	1226	L_1, V
Faiel-2 (LG)	2	4.0	H	15 May	22	1555	833	L_2, P, S
Faiel-3 (LG)	3	5.0	H	26-30April	11	596	154	L_2, P, S
Foz-3 (LG)	3	14.0	L	28 June	7	352	477	L_2, P, S
Valada-PI1-3 (AZ)	3	6.0	H	end April	8	331	22	P, S
Valada-PI2 -3(AZ)	3	3.5	H	middle May	22	1309	154	L_2, P, S
Valada-LQ-3 (AZ)	3	14.5	H	5 April	11	599	110	L_2, P, S
Valada-AB-3 (AZ)	3	5.8	H	19 April	11	603	57	L_2, P, S
Canha-3 (CN)	3	12.0	H	10-14May	13	696	213	L_2, P, S
Coruche1-2 (CR)	2	0.2	L	15 May	7	226	59	P, S
Coruche2-2 (CR)	2	0.2	H	13 May	7	227	134	P, S
Clarianos1-2 (LG)	2	12.0	H	24 May	17	1083	715	V
Clarianos2-3 (LG)	3	10.0	H	middle May	8	426	70	V

[1] Fields location and municipalities: AZ- Reguengos de Valada, Azambuja; CR- Coruche, Coruche; CN- Canha, Montijo, LG- Lezíria Grande, Vila Franca de Xira.

[2] Soils: H - heavy soils from alluvial origin: clays, silts; L=light soils: sandy, sandy loams, loams.

[3] Studies: L_1- egg location (preliminary), L_2-egg location, P-spatial pattern, S-sequential sampling plan; V-validation of the sequential sampling plan.

Table S2. Number of eggs per plant in the margin and the main interior of tomato fields – comparison by Wilcoxon matched pair test (N=observation dates; Z=test statistic; p=significance level).

**Tomato field**	**N**	**Z**	**p**
Faiel-2002	12	-0.235	0.814
Faiel-2004	11	-0.267	0.790
Foz-2004	7	-1.522	0.128
Canha-2004	13	-2.272	0.023
The four fields all together	43	-1.169	0.090

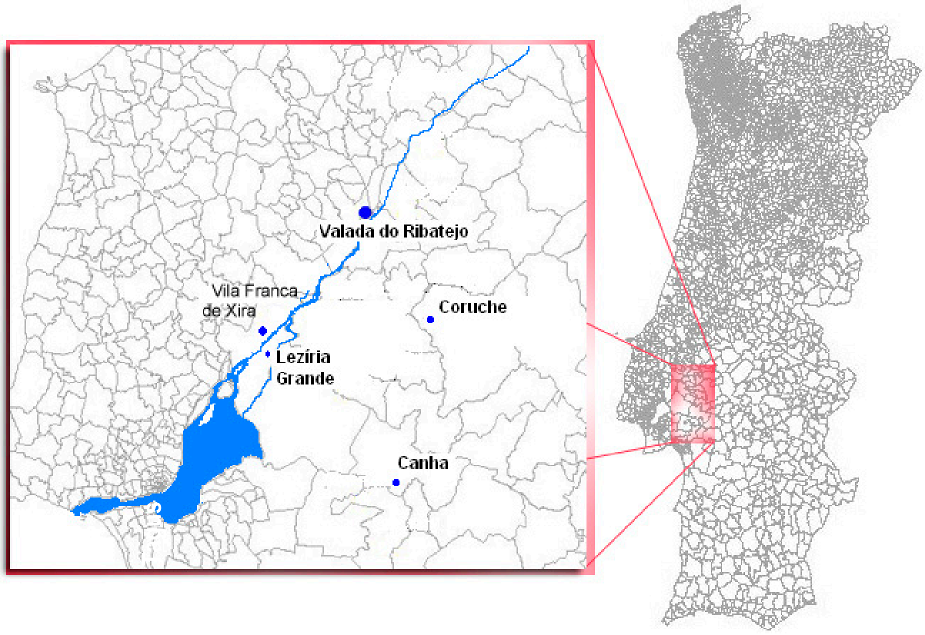

Figure S1. Location of the monitored fields in the Ribatejo region (Portugal).

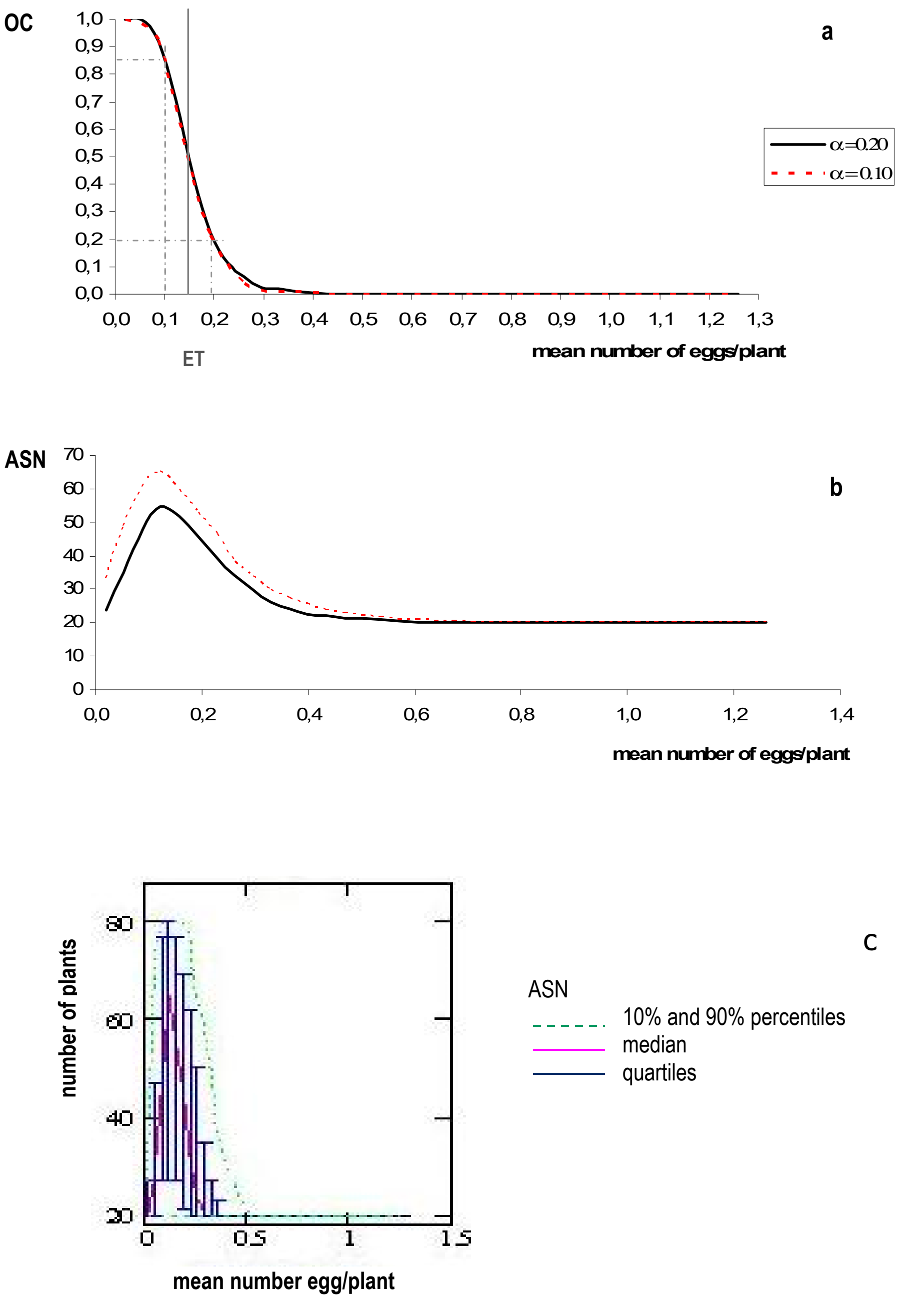

Figure S2. Sequential sampling for risk assessment: OC (a), ASN (b) and ASN quartiles/percentiles (c) curves for the sequential sampling plan presented on Figure 3, estimated by simulation using the validation TPL parameters (Table 3) and Crop Protection Decision-Making Mathcad worksheets of the electronic version of Binns et al .[43] (ET=economic threshold=0.15 egg/plant).

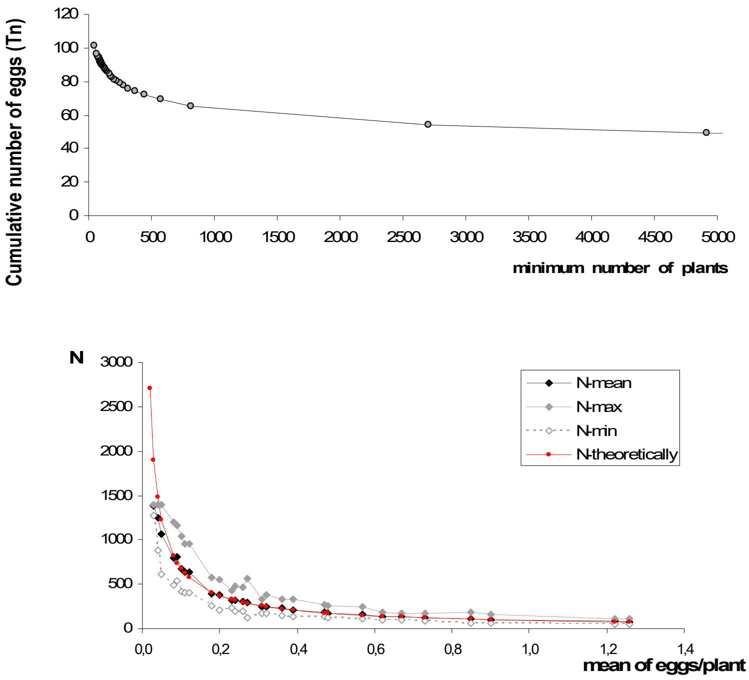

Figure S3. Sequential sampling for density estimation in ecological studies: (a) stop line; (b) mean, maximum and minimum sample sizes resulted from 1000 re-sampling iterations with RVSP2 for fruitworm egg density higher than 0.02 egg/plant, plotted alongside the theoretically calculated sample size (TPL parameters from the model – table 8; validation data sets from validation fields – table 1; sequential sample plan calculated by Green's method with D=0.15; minimum sample size set at 20 plants).
